# Perceived stress and depression among Chinese nurses: a cross-sectional mediation analysis of psychological flexibility and its components

**DOI:** 10.3389/fpsyt.2026.1581857

**Published:** 2026-05-28

**Authors:** Zeyu Huang, Pan Diao, Tian Tian, Lei Yang, Xiaomei Li

**Affiliations:** 1 School of Nursing, Health Science Center, Xi’an Jiaotong University, Xi’an, China; 2 Orthopaedic Center, The Second Affiliated Hospital of Xi’an Jiaotong University, Xi’an, China

**Keywords:** Chinese nurses, depression, mediation analysis, perceived stress, psychological flexibility

## Abstract

**Background:**

Perceived stress is a significant risk factor for depression among healthcare professionals. While this fundamental relationship is well-documented, the potential mediating mechanisms—specifically the role of psychological flexibility and its distinct components—in buffering the impact of perceived stress on depression within the nursing community remain poorly understood.

**Methods:**

This cross-sectional study recruited 3,920 nurses from three tertiary Grade A public hospitals in Xi’an, China, using convenience sampling, of whom 3,611 were included in the final analysis. Data were collected online using the Chinese versions of Cohen’s Perceived Stress Scale and the Comprehensive Assessment of Acceptance and Commitment Therapy Processes, as well as the Patient Health Questionnaire-9. Pearson correlation analyzes and mediation analysis were conducted to examine the associations among perceived stress, psychological flexibility, and depression.

**Results:**

Depression was positively correlated with perceived stress (*r* = 0.63, *p* < 0.01). Mediation analysis indicated that perceived stress was significantly associated with depression both directly (*b* = 0.47, 95% *CI*: 0.45 to 0.49) and indirectly via psychological flexibility (*b* = 0.12, 95% *CI*: 0.11 to 0.13) and its components, including acceptance and cognitive defusion (*b* = 0.14, 95% *CI*: 0.13 to 0.15), and values and committed action (*b* = 0.06, 95% *CI*: 0.04 to 0.08). The indirect association through mindfulness and self-as-context was not significant (*b* = 0.01, 95% *CI*: 0.00 to 0.02).

**Conclusions:**

Psychological flexibility and some of its components were involved in partial indirect associations between perceived stress and depression among Chinese nurses. These findings suggest that psychological flexibility may help understand how perceived stress is statistically associated with depression in this population. Given the cross-sectional design, however, the results should be interpreted as statistical associations rather than evidence of causal relationships.

## 1 Introduction

Nurses are frontline healthcare professionals who experience substantial stress and a considerable burden of depression (1–4). A China-led multicenter cross-sectional study reported that the prevalence of depression among nurses was 26% (5). Depression among nurses warrants attention not only because it affects their own physical and mental health, but also because it has been associated with poorer quality of care, reduced patient safety, and instability in the nursing workforce. Accordingly, addressing depression among nurses has become an increasingly important priority in nursing research and practice.

Perceived stress has long been recognized as an important correlate of depression (6, 7). Among nurses, higher levels of perceived stress have been linked to greater depression, and some studies suggest that perceived stress explains a substantial proportion of the variance in depression (8). Perceived stress tends to increase when individuals appraise external demands as exceeding their available coping resources (9). Even so, the psychological processes through which perceived stress is associated with depression remain insufficiently understood. In this context, examining potential mediating mechanisms between perceived stress and depression among nurses is important, as a better understanding of this association may help identify potential intervention targets and inform the selection of appropriate preventive and supportive strategies.

Psychological flexibility refers to the ability to fully engage in the present moment as a conscious individual and to alter or maintain behavior flexibly to pursue valued goals, especially when faced with unexpected life events (10, 11). It comprises three components: acceptance and cognitive defusion, mindfulness and self-as-context, and value and committed action. Numerous studies have identified that psychological flexibility and its components are correlated with both perceived stress and depression (12–15). Because psychological flexibility is a multidimensional construct (16), its components may not relate to perceived stress and depression in the same way or to the same extent. Examining its individual components may therefore help clarify whether specific aspects of psychological flexibility also show distinct indirect associations in the relationship between perceived stress and depression. Therefore, psychological flexibility and its components may be involved in the indirect associations between perceived stress and depression.

Correlated studies have shown that perceived stress is significantly negatively correlated with psychological flexibility and significantly positively correlated with psychological inflexibility (12, 17). From the perspective of Lazarus’ Stress and Coping Theory (18), individuals under higher perceived stress may be more likely to adopt maladaptive coping responses, which may in turn be related to lower psychological flexibility. Meanwhile, psychological flexibility has been consistently reported to be negatively correlated with depression (15, 19, 20). Higher psychological flexibility has also been linked to more adaptive cognitive-emotional coping, whereas lower psychological flexibility has been associated with greater emotional distress, including depression (21, 22). Together, these findings provide a rationale for examining psychological flexibility as a potential mediator in the association between perceived stress and depression.

The Job Demands–Resources (JD-R) model is a helpful framework for understanding health outcomes in contexts characterized by high job demands (23). The model proposes that high job demands can impose sustained physical and psychological strain, whereas resources can buffer these pressures and facilitate adaptation and recovery (24, 25). In nursing, heavy workloads, professional competency requirements, and complex interpersonal interactions constitute substantial job demands, which may contribute to elevated perceived stress and poorer mental health. Within this framework, psychological flexibility may function as a personal resource relevant to understanding the association between perceived stress and depression. Examining its role may therefore help identify potential intervention targets for nurses.

Based on prior evidence that perceived stress is negatively associated with psychological flexibility and that psychological flexibility is negatively associated with depression, this study examined a mediation model in which psychological flexibility was tested as a potential mediator of the association between perceived stress and depression among nurses. Because psychological flexibility is a multidimensional construct and its components may differ in their associations with perceived stress and depression, its three components—acceptance and cognitive defusion, mindfulness and self-as-context, and values and committed action—were also examined. In addition, gender, age, years of work experience, marital status, number of children, economic status, educational level, and professional title were included as covariates because previous studies have shown that these variables may be associated with depression among nurses and could therefore confound the associations of interest (26–33). We hypothesized that perceived stress would be negatively associated with psychological flexibility and its three components, and that psychological flexibility and these components would be negatively associated with depression. We further hypothesized that psychological flexibility and its three components would show significant indirect effects in the association between perceived stress and depression.

## 2 Methods

### 2.1 Participants and study design

This cross-sectional study was conducted in Xi’an, China, between December 2023 and January 2024. Using convenience sampling, we recruited 3,920 nurses from three tertiary Grade A public general hospitals in Xi’an. All three hospitals were large institutions that integrated clinical care, teaching, and research. The inclusion criteria were as follows: (1) nurses with professional qualification certificates; (2) nurses who provided informed consent and participated voluntarily in the survey. The exclusion criteria were: (1) nurses on leave during the survey period; (2) visiting or rotating nurses; (3) intern nurses. Before the formal survey, the researchers contacted the nursing departments of the three hospitals, introduced the purpose of the study to the directors of nursing, and obtained their cooperation. The head nurses of each department then assisted in informing eligible nurses about the voluntary, unpaid online questionnaire survey. Investigators recorded the number of nurses who voluntarily agreed to participate and met the inclusion criteria, and eligible nurses were invited by the investigators and head nurses to join the corresponding WeChat survey groups for each department in each hospital.

The study was approved by the Human Sciences Ethics Committee of the Xi’an Jiaotong University Health Science Centre (approval number: 2023-1336). The research objectives, the principle of information confidentiality, and the principle of voluntary participation were fully explained to the participants, and electronic informed consent was obtained.

### 2.2 Measurement process

Two trained members of the research team coordinated the online data collection procedure and provided participant support when needed. Before completing the questionnaire, participants were informed of the study purpose, confidentiality principles, and the voluntary nature of participation. After informed consent had been obtained, standardized instructions for completing the questionnaire were provided to participants through their departmental WeChat groups. Subsequently, a Wenjuanxing (a professional online survey platform in China) link was distributed in the corresponding departmental WeChat survey groups of each hospital, and participants completed the questionnaire independently to reduce potential response bias. During the survey period, the investigators were available through WeChat to answer participants’ questions and provide clarification when needed. The estimated time to complete the questionnaire was approximately 20 minutes.

### 2.3 Measures

The research team developed a General Information Questionnaire in accordance with the study objectives. It included two sections: a demographic survey and an occupational factors survey. The demographic survey covered six items: gender, age, marital status, number of children, economic status, and education. The occupational factors survey included two items: working years and professional title.

The Chinese Version of Cohen’s Perceived Stress Scale (CPSS) was designed by Professor Cohen from the Department of Psychology at Carnegie Mellon University based on Lazarus’ stress and coping theory (6). The Chinese version was localized and revised by Chinese scholars, including Yang Tingzhong. It comprised 14 items in two dimensions, tension and loss of control, with seven items in each dimension. Items were rated on a 5-point Likert scale, with response options ranging from never to always. The scale included both forward-scored and reverse-scored items. Items 1, 2, 3, 8, 11, 12, and 14 were scored in the forward direction, whereas items 4, 5, 6, 7, 9, 10, and 13 were reverse scored. The total score was obtained by summing all items and ranged from 0 to 56. Respondents with scores of 25 or above were considered to be under health-risk stress. Internal consistency was assessed using Cronbach’s α, and the overall scale showed a Cronbach’s α of 0.78. The scale had been widely used in China and had demonstrated good validity. It was used to measure perceived stress (predictor) in the present study. The Cronbach’s α coefficient of this scale was 0.91 in the present study.

The Chinese version of the Comprehensive Assessment of Acceptance and Commitment Therapy Processes (CompACT) was initially developed by Francis based on Relational Frame Theory (11). It was later translated and validated by Professor Zhuo-Hong Zhu’s team and colleagues, including Ming Wang, at the Institute of Psychology, Chinese Academy of Sciences. The questionnaire comprised 15 items across three dimensions: acceptance and cognitive defusion, mindfulness and self-as-context, and values and committed action. Internal consistency was evaluated using Cronbach’s α, and the overall scale showed a Cronbach’s α of 0.87, indicating good internal consistency. The scale used a 7-point Likert response format, with options ranging from never to always. In the original scoring procedure, items 4–7 and 9–15 were reverse scored, and the total score was calculated by summing all item scores, with higher total scores indicating lower psychological flexibility. For ease of interpretation, in the present study, items 1, 2, 3, and 8 were reverse scored instead. Specifically, these four items were recoded so that original scores of 0, 1, 2, 3, 4, 5, and 6 were reassigned to 6, 5, 4, 3, 2, 1, and 0, respectively. The recoded scores were then summed with the scores of the remaining items to yield a total score ranging from 0 to 90, such that higher total scores indicated greater psychological flexibility. The CompACT was used to measure psychological flexibility as the mediating variable in the present study. In this study, Cronbach’s α for the scale was 0.84.

The Patient Health Questionnaire-9 (PHQ-9) was developed by Spitzer and colleagues on the basis of the diagnostic criteria in the *Diagnostic and Statistical Manual of Mental Disorders*, Fourth Edition (DSM-IV). It was a self-administered depression module from the full PHQ (34). The questionnaire consisted of nine items, each rated on a 4-point Likert scale. The response categories were not at all, several days, more than half the days, and nearly every day. All items were scored positively. The total score was calculated by summing all item scores and ranged from 0 to 27. A score of 0 ~ 4 indicated no depression, 5 ~ 9 indicated mild depression, 10 ~ 14 indicated moderate depression, 15 ~ 19 indicated moderately severe depression, and 20 ~ 27 indicated severe depression. The questionnaire showed good internal consistency, with a Cronbach’s α of 0.84. It had been widely used internationally and was recognized as a valid instrument. The PHQ-9 was used to measure depression (outcome) in the present study. In this study, the Cronbach’s α coefficient of the scale was 0.96.

### 2.4 Data analysis

The data were collected and downloaded through the Wen Juan Xing online platform. All items were set as required, and incomplete questionnaires could not be submitted. Consequently, there were no missing item data, and no additional missing data handling was performed. Invalid responses were defined as careless responding, including obvious repetitive response patterns and uniform responses across all items. These cases were excluded during the data cleaning stage. To preliminarily assess potential common method bias, Harman’s single-factor test was conducted. SPSS 24.0 was employed to perform statistical analysis on the data. The descriptive data of the main variables were reported using the mean and standard deviation (M ± SD). The independent-samples t tests and one-way ANOVA were used to analyze differences in depression scores across demographic and occupational characteristics among Chinese nurses. For these comparisons, effect sizes were calculated using Cohen’s *d* or *η²* to assess the magnitude of the differences. Formal adjustments for multiple comparisons were not implemented for the independent samples t-tests, as these demographic analyzes were fundamentally exploratory and preliminary. Their primary purpose was to provide a descriptive profile of the sample, rather than to serve as tests of the study’s central hypotheses. Pearson correlation was used for the correlation analysis among variables. To test the mediation effects, the bias-corrected bootstrap method using the SPSS Process macro (Model 4) was utilized. Given the cross-sectional design, the temporal sequence among variables was based on theoretical assumptions rather than empirical verification; therefore, the findings were interpreted as statistical, rather than causal, indirect effects. A total of 5,000 bootstrap samples with replacement were drawn to obtain a 95% confidence interval of the statistical mediating effect value. If the upper and lower limits of the interval did not include 0, the statistical mediating effect was considered statistically significant. Finally, a sensitivity analysis was conducted to assess the robustness of the statistical mediation model. Specifically, the unadjusted models were compared with models adjusted for gender, age, working years, marital status, number of children, economic status, education, and professional title.

## 3 Results

A total of 3,920 questionnaires were distributed online, and 3,611 valid questionnaires were eventually collected, with an effective response rate of 92.12%. The main reasons for nonparticipation included incomplete or incorrectly filled questionnaires. The general demographic characteristics of 3,611 nurses are shown in **Table 1**.

**Table 1:** Demographic and occupational information of participants (n = 3,611).

Variable	n (%)
Gender	
Male	120 (3.30)
Female	3,491 (96.70)
Age	
≤30	1442 (39.90)
30-40	1631 (45.20)
>40	538 (14.90)
Working years	
≤5	2805 (77.70)
5-10	400 (11.10)
10-20	295 (8.20)
>20	111 (3.10)
Marital status	
Unmarried	971 (26.90)
Married	2566 (71.10)
Divorced	74 (2.00)
Number of children	
0	1348 (37.30)
1	1889 (52.30)
≥2	374 (10.40)
Economic status (RMB)	
≤5000	1057 (29.30)
5000-8000	1395 (39.60)
8000-10000	574 (15.90)
10000-15000	391 (10.80)
>15000	194 (5.40)
Education	
Junior college degree or below	545 (15.10)
Bachelor degree or above	3066 (84.90)
Professional title	
Others	3,497 (96.80)
Chief nurse	114 (3.20)

### 3.1 Common method bias

In our study, Harman’s single-factor test was employed to examine common method bias. All the items of variables were subjected to exploratory factor analysis. The analysis extracted eight common factors, and the first principal component accounted for 35.96% of the variance, below the critical threshold of 40%.

### 3.2 Descriptive statistics of the study variables

The mean depression score was 12.05 ± 6.60. A total of 3,076 Chinese nurses (85.18%) scored above the cutoff point between minor depressive symptoms and mild depression (> 4) on the PHQ-9. Among those who screened positive for depression, 908 (29.52%) had mild depression (5 ~ 9 points), 873 (28.38%) had moderate depression (10 ~ 14 points), 869 (28.25%) had moderately severe depression (15 ~ 19 points), and 426 (13.85%) had severe depression (20 ~ 27 points).

Depression scores differed significantly across nurses’ gender (*p* < 0.01), age (*p* < 0.01), working years (*p* < 0.01), marital status (*p* < 0.01), number of children (*p* < 0.01), economic status (*p* < 0.01), and professional title (*p* < 0.01). Details are presented in **Table 2**.

**Table 2:** Comparisons of depression scores across demographic and occupational characteristics (n = 3,611).

Variable	Depression M ± SD	t/F *(p)*	Cohen d/η^2*^	95% confidence interval	
				Lower	Upper
Gender					
Male	10.63 ± 7.50	-2.11 (< 0.01)	-0.21	-0.40	-0.04
Female	12.10 ± 6.57				
Age					
≤30	10.85 ± 6.55	41.70 (< 0.01)	0.02	0.01	0.03
30-40	12.96 ± 6.60				
>40	12.49 ± 6.26				
Working years					
≤5	13.64 ± 6.02	307.70 (< 0.01)	0.20	0.18	0.23
5-10	6.75 ± 5.45				
10-20	6.40 ± 5.53				
>20	5.79 ± 5.27				
Marital status					
Unmarried	11.27 ± 6.59	13.53 (< 0.01)	0.00	0.00	0.01
Married	12.27 ± 6.59				
Divorced	14.57 ± 6.23				
Number of children					
0	11.55 ± 6.60	6.72 (< 0.01)	0.00	0.00	0.01
1	12.41 ± 6.58				
≥2	12.01 ± 6.74				
Economic status (RMB)					
≤5000	11.10 ± 7.03	12.85 (< 0.01)	0.01	0.01	0.02
5000-8000	11.92 ± 6.62				
8000-10000	12.89 ± 6.14				
10000-15000	13.31 ± 5.77				
>15000	13.07 ± 6.13				
Education					
Junior college degree or below	11.77 ± 6.57	1.09 (= 0.30)	0.00	0.00	0.01
Bachelor degree or above	12.10 ± 6.61				
Professional title					
Others	12.11 ± 6.61	3.61 (< 0.01)	0.32	0.13	0.50
Chief nurse	10.03 ± 6.06				

*Cohen’s *d* was reported for binary comparisons, and *η²* was reported for variables with more than two groups. The 95% confidence interval refers to Cohen’s *d* or *η²*.

### 3.3 Correlation analysis

**Table 3** presents the correlations among the study variables. Depression was significantly and positively correlated with perceived stress and significantly negatively correlated with psychological flexibility. Moreover, psychological flexibility was significantly negatively correlated with perceived stress.

**Table 3:** Inter-correlations among measures (n = 3,611).

Variable	PS	PF	D
PS	1.00		
PF	-0.60**	1.00	
D	0.63**	-0.17**	1.00

**p < 0.01. PS, perceived stress; PF, psychological flexibility; D, depression.

### 3.4 Mediation analysis

**Figure 1:**
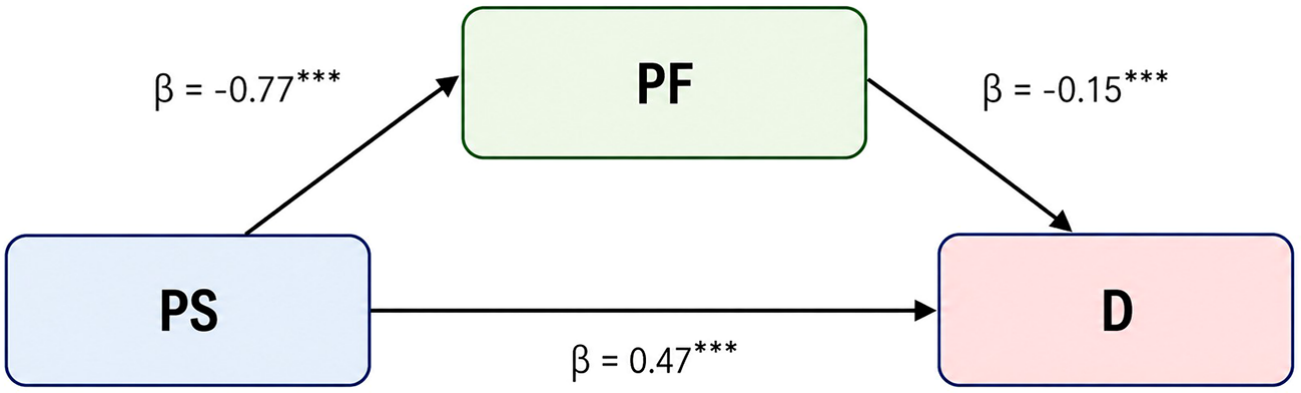
Mediation model exploring the indirect effects of psychological flexibility between perceived stress and depression after adjusting for covariates. ***P < 0.001. PS, perceived stress; PF, psychological flexibility; D, depression.

**Figure 2:**
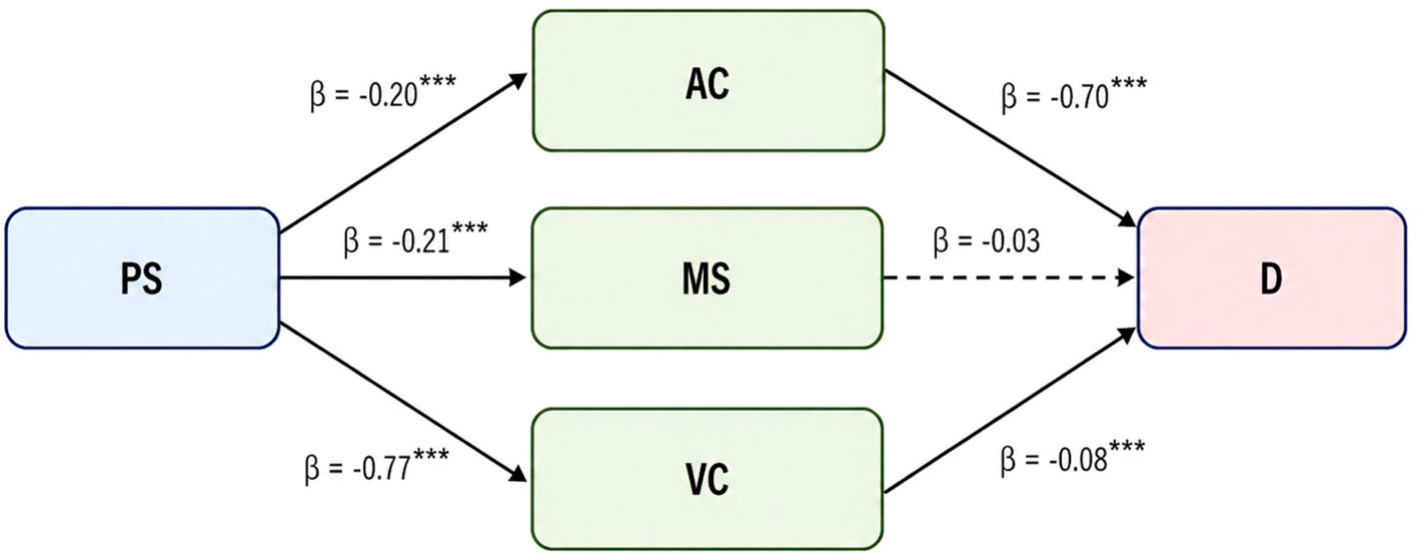
Mediation model exploring the indirect effects of the components of psychological flexibility between perceived stress and depression after adjusting for covariates. ***P < 0.001. PS, perceived stress; PF, psychological flexibility; D, depression; AC, acceptance and cognitive defusion; MS, mindfulness and self-as-context; VC, values and committed action.

We examined the mediating role of psychological flexibility in the association between perceived stress and depression. Sensitivity analyzes indicated that the overall pattern of results was materially unchanged after adjustment for covariates. In the unadjusted models, perceived stress scores were significantly negatively associated with psychological flexibility and all of its component scores. Psychological flexibility and its components, including acceptance and cognitive defusion, and values and committed action, were significantly and negatively associated with depression scores. However, the association between mindfulness and self-as-context and depression was not statistically significant (**Table 4**). After adjustment for covariates, the overall pattern of associations remained largely unchanged, and the previously significant associations remained statistically significant (**Figures 1**, **2**; **Table 5**).

**Table 4:** Indirect effects and 95% confidence intervals without covariate adjustment.

Pathway	Estimate	Estimate (standardized)	95% confidence interval	
			Lower	Upper
PS-PF-D	0.12	0.19	0.11	0.13
PS-AC-D	0.14	0.22	0.13	0.15
PS-MS-D	0.01	0.01	0.00	0.02
PS-VC-D	0.06	0.10	0.05	0.08

PS, perceived stress; PF, psychological flexibility; AC, acceptance and cognitive defusion; MS, mindfulness and self-as-context; VC, values and committed action; D, depression.

**Table 5:** Indirect effects and 95% confidence intervals with covariate adjustment.

Pathway	Estimate	Estimate (standardized)	95% confidence interval	
			Lower	Upper
PS-PF-D	0.12	0.19	0.11	0.13
PS-AC-D	0.14	0.22	0.13	0.15
PS-MS-D	0.01	0.01	0.00	0.02
PS-VC-D	0.06	0.09	0.04	0.08

PS, perceived stress; PF, psychological flexibility; AC, acceptance and cognitive defusion; MS, mindfulness and self-as-context; VC, values and committed action; D, depression.

As shown in **Figure 1**, the unstandardized direct effect of perceived stress on depression was 0.47. The path coefficient for the association between perceived stress and psychological flexibility was −0.77, whereas that for the association between psychological flexibility and depression was −0.15. The resulting unstandardized indirect effect via psychological flexibility was approximately 0.12 [(−0.77)×(−0.15)≈0.12]. Given a total effect of 0.59, the indirect effect accounted for approximately 20.34% of the total effect. These findings suggest that the indirect association through psychological flexibility accounted for approximately one-fifth of the overall association between perceived stress and depression. We further examined the unstandardized indirect effects of the three components of psychological flexibility in the association between perceived stress and depression after adjustment for covariates. As shown in **Figure 2**, perceived stress was significantly negatively associated with acceptance and cognitive defusion (β = −0.20), mindfulness and self-as-context (β = −0.21), and values and committed action (β = −0.77). In turn, acceptance and cognitive defusion (β = −0.70) and values and committed action (β = −0.08) were significantly and negatively associated with depression, whereas mindfulness and self-as-context was not significantly associated with depression (β = −0.03).

As presented in **Table 5**, both the unstandardized and standardized indirect effects of psychological flexibility, acceptance and cognitive defusion, and values and committed action were statistically significant after adjustment for covariates. In contrast, neither the unstandardized nor the standardized indirect effect of mindfulness and self-as-context was statistically significant. Moreover, as shown in **Figure 1** and **2**, both the unstandardized and standardized direct effects of perceived stress on depression remained statistically significant after the mediators were included in the model. Taken together, these findings suggest that psychological flexibility, acceptance and cognitive defusion, and values and committed action were involved in significant partial indirect associations between perceived stress and depression, whereas mindfulness and self-as-context was not.

## 4 Discussion

The principal findings of this study indicated that perceived stress was associated with depression both directly and indirectly through psychological flexibility and some of its components. These findings suggest that psychological flexibility may be relevant to understanding how perceived stress is statistically associated with depression among Chinese nurses.

In the present study, 85.18% of participants scored above the PHQ-9 threshold for at least mild depression, a proportion comparable to that reported by Peng et al. among 452 Chinese nurses (83.0%) (35). This finding suggests that depression may be common among Chinese nurses. However, the observed rate should be interpreted with caution. First, the cutoff score used in this study was > 4, which corresponds to the threshold between minimal and mild depression and functions as a sensitive screening criterion rather than a diagnostic indicator of clinical depression. This relatively low threshold may have increased the proportion of participants classified as screening positive. Second, the sample was drawn from tertiary hospitals, where nurses are often exposed to heavy workloads and high occupational stress, both of which may be associated with a higher level of depression. Third, because participation was voluntary and based on an online survey, response bias cannot be excluded; nurses who were more concerned about their psychological condition may have been more likely to participate. Therefore, the present findings are more appropriately interpreted as reflecting the proportion of nurses in this sample who reported at least mild depression, rather than the prevalence of clinical depression among Chinese nurses more generally.

In the present study, depression scores differed significantly across gender, age, working years, marital status, number of children, economic status, and professional title. Most of these effects, however, were small in magnitude, suggesting that although subgroup differences were statistically detectable, their practical importance was generally limited. Among these variables, working years showed the largest effect size, indicating that career stage may be more closely associated with depression than other demographic and occupational characteristics in this sample. Female nurses reported higher depression scores than male nurses, a pattern broadly consistent with previous studies showing that depression is more commonly reported in women (25). This difference may be related to the combined influence of occupational burden and gender-related social roles, although the small effect size suggests that its practical magnitude was limited (36, 37). This finding should nevertheless be interpreted with caution, because male participants accounted for only 3.3% of the sample, and this marked gender imbalance may have limited the stability of the comparison.

Depression scores were also higher among nurses older than 30 years, particularly those aged 30–40 years. One possible explanation is that nurses in this stage of life often face overlapping demands from clinical work, career development, and family responsibilities, all of which may be associated with greater emotional strain (38, 39).

Among the significant factors examined, working years showed the largest effect size. Nurses with fewer years of work experience reported substantially higher depression scores than those with more years of experience. This pattern may suggest that the early stage of nursing practice is associated with greater psychological vulnerability, as nurses at this stage are often still adapting to demanding clinical environments and may have less developed coping resources (40–42). An alternative explanation is that nurses with greater psychological distress may be less likely to remain in long-term clinical practice, which could partly contribute to the observed gradient across working-year groups.

Marital status was also significantly associated with depression, with divorced nurses showing the highest scores. This pattern may be related to the emotional strain and reduced social support that can accompany marital disruption (43).

Nurses with children also tended to report somewhat higher depression scores than those without children. Although the effect size was small and the pattern was not strictly linear, parenting responsibilities may be associated with greater work–family conflict and fewer opportunities for rest and recovery (38).

Higher-income groups also showed higher depression scores. In this sample, this pattern may suggest that higher income is linked to positions involving greater responsibility, heavier workloads, or more complex demands, rather than simply indicating better material resources (44).

Professional title was likewise associated with depression, with chief nurses reporting lower scores than nurses with lower professional titles. One possible explanation is that greater experience, clearer role expectations, and stronger decision latitude may be related to better management of occupational stress among senior nurses (24, 45).

Taken together, these findings suggest that depression among Chinese nurses vary across demographic and occupational subgroups, although most of the observed differences were small in magnitude. Among the factors examined, working years showed the largest effect size, suggesting that early-career nurses may warrant particular attention in mental health support. Nevertheless, these subgroup differences should be interpreted as descriptive associations rather than evidence of causal effects.

In the present study, perceived stress was positively correlated with depression, suggesting that nurses who reported higher perceived stress also tended to report more severe depression. This finding is consistent with previous studies (46–48). Perceived stress reflects individuals’ appraisal of stressfulness, including feelings of tension and loss of control (6), both of which have been linked to depression in prior research. Previous studies have also shown that higher perceived stress is associated with depression and other adverse mental health outcomes (3, 4). Similarly, studies in nursing populations have consistently shown that elevated perceived stress is related to higher levels of depression (2, 49–52).

In this study, psychological flexibility was negatively correlated with depression, suggesting that greater psychological flexibility was associated with lower levels of depression. Within the ACT framework, psychological flexibility refers to the capacity to accept internal experiences, remain present, and act in accordance with personal values despite distress (15, 53). This capacity may be relevant to understanding more adaptive responses to stressors and, consequently, better mental health outcomes. The present finding is consistent with previous evidence showing that lower psychological flexibility is associated with higher levels of depression (54–56).

Our findings indicate that psychological flexibility and its components were involved in partial indirect associations between perceived stress and depression among Chinese nurses. However, given the cross-sectional design, causal inferences cannot be drawn. Future longitudinal or interventional studies are needed to clarify these associations and examine their temporal ordering.

Psychological flexibility was involved in a significant indirect association between perceived stress and depression among Chinese nurses. This finding suggests that psychological flexibility may be relevant to understanding how perceived stress is statistically associated with depression in this population. Viewed through the lens of Lazarus’ Stress and Coping Theory (57), psychological flexibility shares conceptual overlaps with adaptive coping strategies. When facing occupational stressors, individuals with lower psychological flexibility may be more likely to rely on emotion-focused coping, including experiential avoidance, rumination, or self-blame, rather than active problem-solving. These maladaptive cognitive patterns may help explain the observed association between higher perceived stress and greater depression. Previous research has also suggested that some Chinese nurses tend to adopt emotion-focused coping strategies in stressful clinical environments (58), which may be relevant to understanding higher levels of psychological distress. Conversely, nurses with higher psychological flexibility may be more likely to adopt more adaptive coping responses (59), which may help explain why greater psychological flexibility was statistically associated with lower depression scores in the present study.

The acceptance and cognitive defusion component of psychological flexibility also appeared to be involved in the indirect association between perceived stress and depression among Chinese nurses. Drawing on the concept of experiential avoidance, rigid attempts to control or suppress psychological distress may paradoxically intensify such experiences rather than reduce them (60). In the clinical context, nurses with lower levels of acceptance toward occupational difficulties may be more prone to cognitive fusion, in which negative thoughts are treated as literal truths rather than transient mental events. This cognitive rigidity may limit adaptive responses to present-moment experiences. Consequently, reduced coping flexibility may be associated with more negative self-evaluative processing and higher depression levels.

The values and committed action component of psychological flexibility also appeared to be involved in the indirect association between perceived stress and depression among Chinese nurses. This finding suggests that maintaining alignment with personal and professional values, together with goal-directed action, may be relevant to understanding lower depression under conditions of occupational stress. Consistent with principles from positive psychology (61), value-congruent behavior may be associated with greater psychological resilience and lower distress. In clinical practice, nurses whose professional values are more closely aligned with their personal beliefs may be more likely to adopt proactive responses when encountering workplace adversity. For example, individuals guided by values such as helping others may be more likely to engage in meaningful actions, such as improving patient care routines or seeking constructive support from colleagues, even under conditions of high occupational stress. Continuous engagement in value-driven action may help explain why this component was statistically linked to lower depression in the present study.

Interestingly, the mindfulness and self-as-context component of psychological flexibility did not show a significant indirect association between perceived stress and depression among Chinese nurses. This non-significant finding may be understood in relation to the high-intensity clinical environment of Chinese tertiary hospitals. Attentional Resources Theory suggests that cognitive capacity is limited (62). Under conditions of acute workload and high urgency (63), where nurses must simultaneously manage emergency care and continuous patient monitoring, attentional resources may be predominantly directed toward external clinical demands. As a result, fewer cognitive resources may remain available for sustained present-moment awareness or metacognitive distancing. In addition, the non-significant result may partly reflect limited measurement sensitivity (64, 65). The items assessing mindfulness and self-as-context involve relatively abstract metacognitive experiences, such as distinguishing the “observing self” from transient thoughts, which may be difficult for nurses in chronically stressful environments to identify and report accurately using self-report measures. Therefore, the lack of statistical significance may reflect limited measurement sensitivity rather than the definitive absence of these psychological processes. Future studies using more context-sensitive methods are needed to further clarify this finding.

## 5 Limitations

Several limitations of the present study should be acknowledged. First, regarding the study design and data collection, the cross-sectional nature of the data precludes any definitive causal inferences regarding the directional mediation of psychological flexibility. While our proposed model is theoretically grounded, future longitudinal or cross-lagged panel studies are required to verify these temporal relationships (66). Furthermore, because all data were collected via self-report questionnaires simultaneously, the results are susceptible to common-method bias and social desirability bias, which may artificially inflate the observed associations between variables. Second, the online recruitment method inherently introduces the risk of self-selection bias. Nurses experiencing higher levels of perceived stress, or those more actively concerned about their mental health, might have been more motivated to complete the survey. This may, in turn, have led to an overestimation of the prevalence of depression in the current sample. In addition, the generalizability of the findings is constrained by the sampling scope and demographic characteristics. The study sample was drawn exclusively from tertiary Grade A hospitals within a single city and exhibited a severely skewed gender distribution. Given the substantial cultural, economic, and organizational differences across various regions and healthcare tiers in China, these findings may not adequately reflect the psychological status of the broader Chinese nursing population. Moreover, although the adjusted models included several demographic and occupational variables, the available covariates were still incomplete, and future studies should incorporate more detailed occupational, psychosocial, and mental health history variables. Future multicenter studies across diverse geographic and clinical settings are warranted to improve sample representativeness.

## 6 Conclusions

In conclusion, psychological flexibility and its components—acceptance and cognitive defusion, as well as values and committed action—were involved in partial indirect associations between perceived stress and depression among Chinese nurses. Given the cross-sectional design and sample characteristics, causal inferences cannot be drawn, and the findings should be interpreted with caution.

These findings may help inform future screening and intervention development in nursing settings. Nursing administrators may consider routine psychological flexibility screening, along with targeted psychological support and ACT-based training, as potentially useful approaches to promoting nurses’ mental well-being and addressing depression.

## Data Availability

The datasets [The mediating effect of psychological flexibility between perceived stress and depression among Chinese nurses: A cross-sectional study] for this study can be found in the [Mendeley Data] (http://doi.org/10.17632/bk5f562x5w.1).
